# Profiling microRNA expression during fracture healing

**DOI:** 10.1186/s12891-016-0931-0

**Published:** 2016-02-16

**Authors:** Takahiro Waki, Sang Yang Lee, Takahiro Niikura, Takashi Iwakura, Yoshihiro Dogaki, Etsuko Okumachi, Keisuke Oe, Ryosuke Kuroda, Masahiro Kurosaka

**Affiliations:** Department of Orthopaedic Surgery, Kobe University Graduate School of Medicine, 7-5-1 Kusunoki-cho, Chuo-ku, Kobe, 650-0017 Japan

**Keywords:** Bone, Fracture healing, microRNA, Microarray

## Abstract

**Background:**

The discovery of microRNA (miRNA) has revealed a novel type of regulatory control for gene expression. Increasing evidence suggests that miRNA regulates chondrocyte, osteoblast, and osteoclast differentiation and function, indicating miRNA as key regulators of bone formation, resorption, remodeling, and repair. We hypothesized that the functions of certain miRNAs and changes to their expression pattern may play crucial roles during the process of fracture healing.

**Methods:**

Standard healing fractures and unhealing fractures produced by periosteal cauterization at the fracture site were created in femurs of seventy rats, with half assigned to the standard healing fracture group and half assigned to the nonunion group. At post-fracture days 3, 7, 10, 14, 21, and 28, total RNA including miRNA was extracted from the newly generated tissue at the fracture site. Microarray analysis was performed with miRNA samples from each group on post-fracture day 14. For further analysis, we selected highly up-regulated five miRNAs in the standard healing fracture group from the microarray data. Real-time PCR was performed with miRNA samples at each time point above mentioned to compare the expression levels of the selected miRNAs between standard healing fractures and unhealing fractures and investigate their time-course changes.

**Results:**

Microarray and real-time polymerase chain reaction (PCR) analyses on day 14 revealed that five miRNAs, miR-140-3p, miR-140-5p, miR-181a-5p, miR-181d-5p, and miR-451a, were significantly highly expressed in standard healing fractures compared with unhealing fractures. Real-time PCR analysis further revealed that in standard healing fractures, the expression of all five of these miRNAs peaked on day 14 and declined thereafter.

**Conclusion:**

Our results suggest that the five miRNAs identified using microarray and real-time PCR analyses may play important roles during fracture healing. These findings provide valuable information to further understand the molecular mechanism of fracture healing and may lead to the development of miRNA-based tissue engineering strategies to promote fracture healing.

## Background

There are more than 18.3 million fractures treated in the United States annually [1]. It has been estimated that 5–10 % of fractures have delayed or disrupted healing, resulting in significant morbidity and tremendous loss of productivity and income [2]. Impairment of fracture healing causes not only individual but also economic damage. In the United States, in 2007, fracture patients who required hospitalization produced over $29 billion in hospital costs [1]. Thus, enhancing quality and/or speed of fracture healing would be of substantial benefit to patients and would reduce the social burden. Such improvement can be realized through a better understanding of fracture healing. Hence, by means of in vitro and in vivo experiments, considerable efforts have been made to elucidate the molecular mechanisms of fracture healing [3–5].

The recent discovery of microRNA (miRNA) has revealed a novel type of regulatory control for gene expression. miRNA is a class of small non-coding RNA that regulates gene expression by binding the 3′-untranslated region (3′-UTR) of target messenger RNAs (mRNAs), leading to inhibition of translation or mRNA degradation [6]. miRNA plays key roles in biological processes, such as cell growth, differentiation, and organ development [7]. Furthermore, miRNA has been associated with human disease, including cancer [8], and, therefore, have potential as novel therapeutic targets. In the field of skeletal biology, several in vitro and a few in vivo studies suggest that miRNA regulates chondrocyte, osteoblast, and osteoclast differentiation and function, indicating that miRNA is a key regulator of bone formation, resorption, remodeling, and repair [9, 10]. miRNA has also been implicated in bone-related disorders, such as osteoporosis [10, 11].

It has recently been reported that miRNAs may contribute to fracture healing. Murata et al. demonstrated that inhibition of miR-92a enhances fracture healing in mice [12]. Seeliger et al. profiled miRNAs in bone tissue from patients with osteoporotic fractures and identified five miRNAs, miR-21, miR-23a, miR-24, miR-100, and miR-125b that are highly associated with osteoporotic fractures [13]. With regard to fracture nonunion, we recently profiled miRNAs in nonunion of the rat femur and identified five miRNAs, miR-31a-3p, miR-31a-5p, miR-146a-5p, miR-146b-5p, and miR- 223-3p that were associated with the development of nonunion [14]. However, the role of miRNAs during fracture healing is not well understood. The elucidation of miRNA expression changes during the process of fracture healing may provide more accurate insight into the molecular pathways that regulate bone repair and regeneration and may inform therapeutic intervention to accelerate fracture healing.

We hypothesized that the functions of certain miRNAs and changes to their expression pattern may play crucial roles during the process of fracture healing. The purpose of this study was to examine miRNA expression profiles during standard fracture healing of the rat femur by microarray analysis and to elucidate the dynamic expression patterns of highly expressed miRNAs during fracture healing.

## Methods

### Femoral fracture animal model

**Fig. 1:**
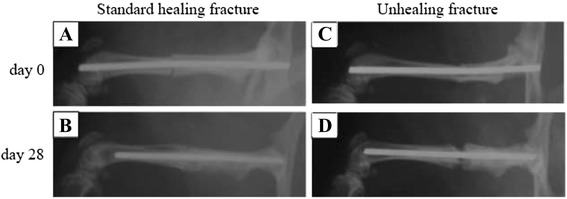
Radiographs of the standard healing fractures (**a** and **b**) and unhealing fractures (**c** and **d**) obtained immediately post-fracture (**a** and **c**) and at post-fracture day 28 (**b** and **d**)

Twelve-week-old male Sprague–Dawley rats (CLEA Japan, Tokyo, Japan) weighing 382.7 ± 6.3 g were used in this study. All animal procedures were performed under the approval and guidance of the Animal Care and Use Committee of Kobe University Graduate School of Medicine. Animals were randomized to receive either a surgical treatment that produces a standard, closed femoral shaft fracture that is known to successfully heal or an unhealing femoral shaft fracture (Fig. 1). The details of these procedures have been previously described [15, 16]. Briefly, a 1.2-mm diameter K-wire was inserted retrograde into the right femoral intramedullary canal and a closed transverse femoral shaft fracture was produced in all animals using a three-point bending apparatus with a drop weight [15]. Hypodermic injection of buprenorphine provided postoperative analgesia. To create unhealing femoral fractures, the fractured site was then exposed through a lateral approach and the periosteum was cauterized circumferentially for a distance of 2 mm on each side of the fracture. This experimental model reproducibly leads to an atrophic nonunion at 8 weeks after surgery [16]. Unprotected weight bearing was allowed post-operatively. Seventy animals were used in this study, with half assigned to the standard healing fracture group and half assigned to the unhealing fracture group. Five animals from each group were euthanized on post-fracture day 14 for microarray analysis and five animals from each group were euthanized on post-fracture days 3, 7, 10, 14, 21, and 28 for real-time polymerase chain reaction (PCR) analysis. These time points were selected based on our previous experiments [17, 18]. At these time points, the newly generated tissues, that is, the fracture callus for the standard healing fractures and the fibrous tissue surrounding the fracture site for the unhealing fractures, were harvested. Following euthanasia, the surrounding muscles were cleanly dissected away from the callus or fibrous tissue generated at the fracture site. The external callus or fibrous tissue generated at the fracture site was excised circumferentially from the underlying intact cortical bone by dissection with a scalpel and rongeur. Care was taken to harvest only newly generated tissue and to not include any underlying intact bone. Because there was no organized callus on post-fracture day 3, the tissue harvested from each model was the fracture hematoma existing at the fracture site.

### miRNA microarray analysis

For miRNA microarray analysis, tissues were harvested on post-fracture day 14 and stored in RNAlater (Ambion, Austin, TX, USA). Total RNA, including miRNA, was extracted from the tissue specimens of five different animals in each group using a miRCURY RNA Isolation Kit-Tissue (Exiqon, Vedbaek, Denmark). The quality of the total RNA was verified using an Agilent 2100 Bioanalyzer profile (Agilent Technologies, Santa Clara, CA, USA). One microgram of total RNA from sample and reference RNAs was labeled with Hy3 and Hy5 fluorescent labels, respectively, using a miRCURY LNA microRNA Hi-Power Labeling Kit (Exiqon). The Hy3-labeled samples and a Hy5-labeled reference RNA sample were mixed pair-wise and hybridized to a miRCURY LNA Array, version 6^th^ Generation (Exiqon), which contains capture probes targeting all human, mouse, and rat miRNAs registered in miRBASE version 16.0. Labeling, hybridization, washing, and scanning were performed following the instructions of the manufacturers. Data analysis was carried out using Feature Extraction 10.7.3.1 (Agilent Technologies).

### Quantitative real-time PCR analysis

For further analysis of the array results, we selected miRNAs that were highly up-regulated in the standard healing fracture group. To compare the expression levels of the selected miRNAs between standard healing fractures and unhealing fractures and to investigate their changes in expression over time in standard healing fractures, real-time PCR was performed on RNA from tissue specimens collected on post-fracture days 3, 7, 10, 14, 21, and 28 (*n* = 5 in each group at each time point). Tissue specimens were homogenized with a T 18 ULTRA-TURRAX homogenizer (IKA Werke, Staufen, Germany) and total RNA, including miRNAs, was extracted using a miRCURY RNA Isolation Kit-Tissue. RNA used for real-time PCR assays did not include any of the RNA used in the microarray assay. Total RNA was reverse transcribed into single-strand cDNA using the miRCURY LNA Universal RT microRNA PCR kit (Exiqon). Real-time PCR analysis was performed in duplicate with a StepOne Sequence Detector (Applied Biosystems, Branchburg, NJ, USA), using SYBR Green master mix and microRNA LNA PCR primer sets (both from Exiqon). U6 was used as an internal control to normalize differences in miRNA levels in each sample. The relative abundance of each miRNA was calculated using the comparative ΔΔCT method, and is presented as the fold change relative to levels in the post-fracture day 3, standard healing fracture sample.

### Statistical analysis

All the quantitative data are presented as means ± standard errors. The values of standard healing fractures and unhealing fractures were compared at each time point using the Mann–Whitney *U*-test. The Kruskal–Wallis test and Mann–Whitney *U*-test with Bonferroni correction were used to compare between time points in the standard healing fractures group. A *p*-value of **<** 0.05 was defined as statistically significant.

## Results

### miRNA microarray analysis

Using a miRNA microarray approach, we tested the expression of 680 rat miRNAs. There were 317 miRNAs that were expressed more highly in standard healing fractures compared with unhealing fractures. From these 317 miRNAs, those that were highly up-regulated were extracted by filtering with a fold change of > 2.0, a low coefficient variation (<50 %), and a high Hy3 signal (>10). With these criteria we identified eight miRNAs: miR-140-3p, miR-140-5p, miR-181a-5p, miR-181d-5p, miR-208b-3p, miR-451a, miR-743b-5p, and miR-879-3p (Table 1). The microarray data have been deposited in the NCBI’s Gene Expression Omnibus (GEO) and are accessible through GEO series accession no. GSE 55088.

**Table 1:** Highly up-regulated miRNAs in standard healing fractures compared with expression in unhealing fractures on post-fracture day 14

miRNA	Fold change Standard healing fracture /Unhealing fracture
miR-181d-5p miR-181a-5p miR-140-5p miR-451a miR-208b-3p miR-743b-5p miR-879-3p miR-140-3p	3.57 3.03 2.86 2.63 2.44 2.33 2.17 2.13

### Real-time PCR analysis

**Fig. 2:**
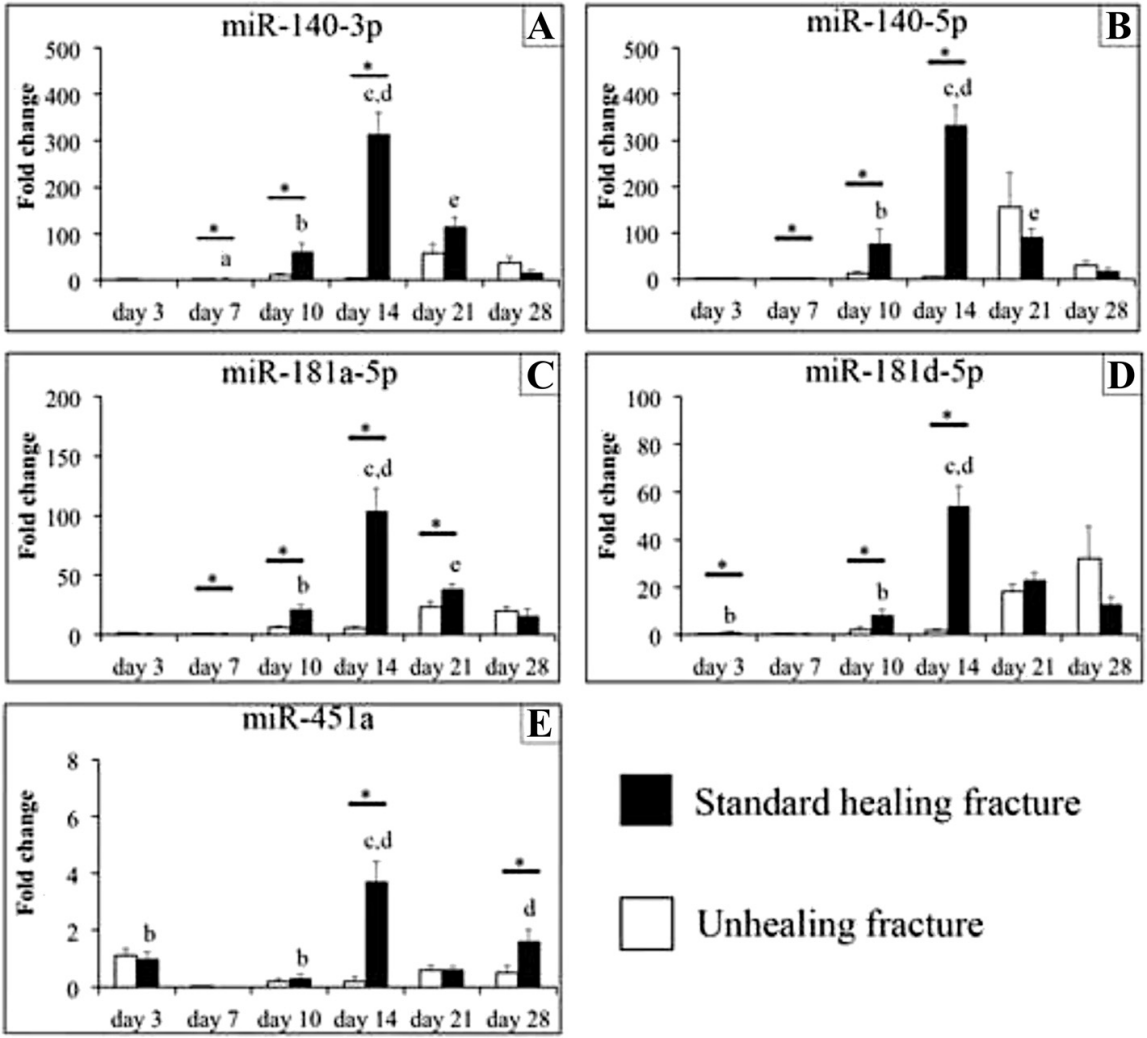
Expression of miR-140-3p (**a**), miR-140-5p (**b**), miR-181a-5p (**c**), miR-181d-5p (**d**), and miR-451a (**e**) in standard healing fractures (solid bars) and in unhealing fractures (blank bars) on post-fracture days 3 and 7, as analyzed by real-time PCR. All graphs show the fold change in expression when expression in the standard healing fracture on day 3 was normalized as 1. Values are the mean ± standard error (*n* = 5 in each group at each time point). *p < 0.05 for indicated groups. a, *p* < 0.05 versus values on day 3 in standard healing fracture; b, *p* < 0.05 versus values on day 7 in standard healing fracture; c, *p* < 0.05 versus values on day 10 in standard healing fracture; d, *p* < 0.05 versus values on day 21 in standard healing fracture; e, *p* < 0.05 versus values on day 28 in standard healing fracture

**Fig. 3:**
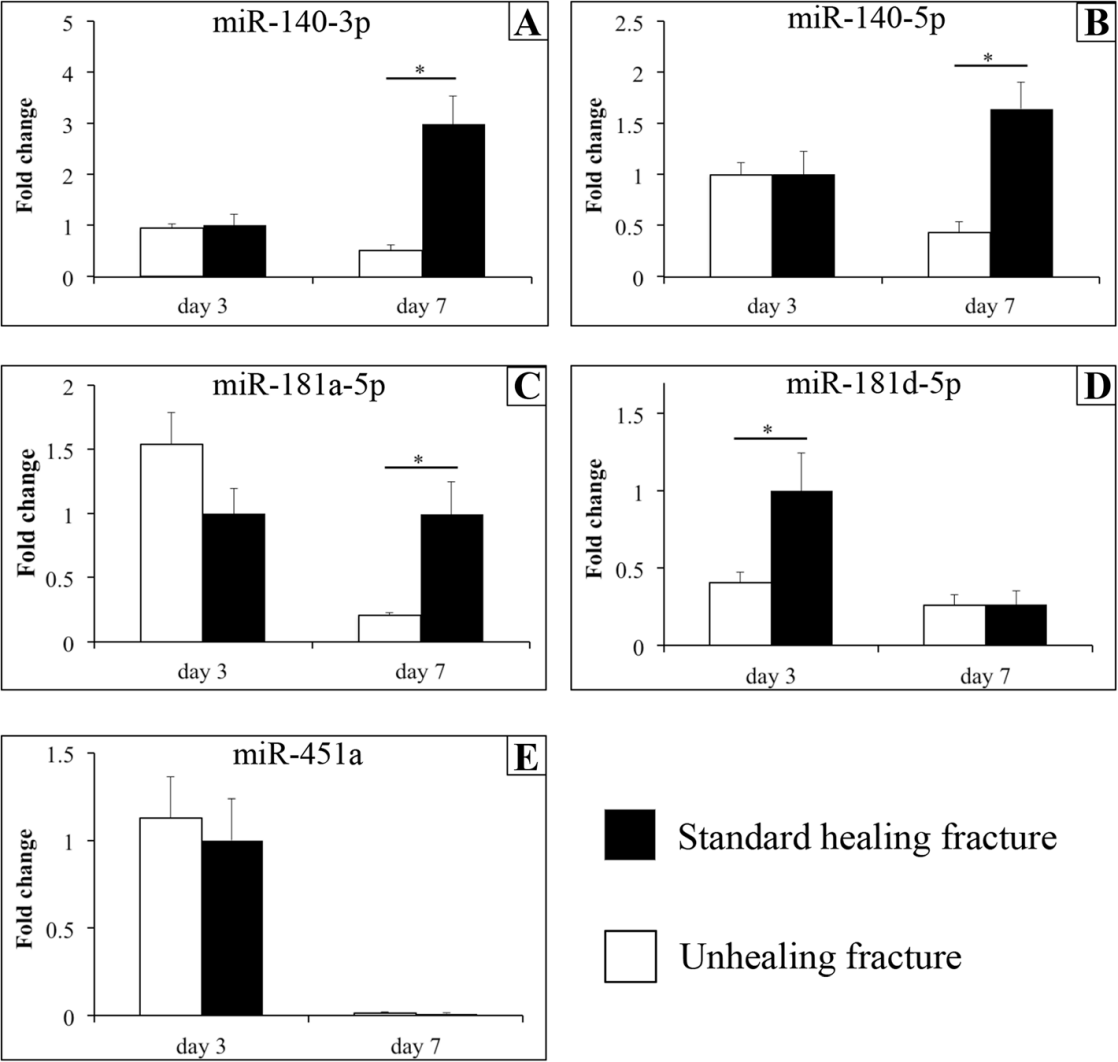
Expression of miR-140-3p (**a**), miR-140-5p (**b**), miR-181a-5p (**c**), miR-181d-5p (**d**), and miR-451a (**e**) in standard healing fractures (solid bars) and in unhealing fractures (blank bars) on post-fracture days 3 and 7, as analyzed by real-time PCR. All graphs show the fold change in expression when expression in the standard healing fracture on day 3 was normalized as 1. Values are the mean ± standard error (*n* = 5 in each group at each time point)

The five most highly expressed miRNAs that were conserved between rat and human were selected from the microarray data. We selected miRNAs that were conserved between rat and human so that our results can reflect standard fracture healing in humans. These miRNAs were miR-140-3p, miR-140-5p, miR-181a-5p, miR-181d-5p, and miR-451a. Validation by real-time PCR analysis confirmed that the expression of all five miRNAs was significantly higher in standard healing fractures compared with unhealing fractures on post-fracture day 14 in a manner consistent with the data from the microarray analysis (Fig. 2, 3; *p* < 0.05). In addition, we followed the dynamic expression patterns of these five miRNAs, as described below.

### miR-140-3p and miR-140-5p

The expression of miR-140-3p and -5p was significantly higher in standard healing fractures than in unhealing fractures on post-fracture days 7, 10, and 14 (*p* < 0.05) (Figs. 2a, b and 3a, b). In standard healing fractures, expression of both increased with time until day 14, and then declined. There were significant differences between days 3 and 7, 7 and 10, 10 and 14, 14 and 21, and 21 and 28 in the expression of miR-140-3p (*p* < 0.05), whereas there were significant differences between days 7 and 10, 10 and 14, 14 and 21, and 21 and 28 in the expression of miR-140-5p (*p* < 0.05).

### miR-181a-5p

The expression of miR-181a-5p was significantly higher in standard healing fractures than in unhealing fractures on post-fracture days 7, 10, 14, and 21 (*p* < 0.05) (Figs. 2c and 3c). Expression in standard healing fractures peaked on day 14 and then declined with time. There were significant differences between days 7 and 10, 10 and 14, 14 and 21, and 21 and 28 (*p* < 0.05).

### miR-181d-5p

The expression of miR-181d-5p was significantly higher in standard healing fractures than in unhealing fractures on post-fracture days 3, 10, and 14 (*p* < 0.05) (Fig. 2d and 3d). The expression in standard healing fractures was declined on day 7, then increased until day 14, and then declined with time. There were significant differences between days 3 and 7, 7 and 10, 10 and 14, and 14 and 21 (*p* < 0.05).

### miR-451a

The expression of miR-451a was significantly higher in standard healing fractures compared with that in unhealing fractures on post-fracture days 14 and 28 (*p* < 0.05) (Figs. 2e and 3e). In standard healing fractures, expression was significantly declined on day 7, then increased until day 14, then again declined on day 21, and then again increased on day 28. There were significant differences between days 3 and 7, 7 and 10, 10 and 14, and 14 and 21, and 21 and 28 (*p* < 0.05).

## Discussion

miRNAs play critical roles in many physiological and pathophysiological process [6], and have recently emerged as key regulators in bone metabolism, development and repair, and in bone-related disease [10–13]. To better understand the underlying physiological mechanisms of fracture healing, the present study focused on miRNA expression profiles in standard healing fractures of the rat femur. This study identified, for the first time, that five miRNAs, miR-140-3p, miR-140-5p, miR-181a-5p, miR-181d-5p, and miR-451a, are highly expressed with dynamic expression patterns in standard healing fractures in rats. These findings potentially give insight into miRNA regulation during fracture healing.

Fracture healing is a complex physiological process that involves a well-orchestrated series of biological events, including inflammation, intramembranous ossification, chondrogenesis, endochondral ossification, and remodeling [3, 4]. This process can be divided into three overlapping phases: inflammation, repair, and remodeling. In the current study, the time point of post-fracture day 14 was selected for microarray analysis because this day is representative of key cellular events in rat fracture healing. Post-fracture day 14 corresponds to the repair phase, which is recognized as a time point representing both soft and hard callus formation, including both intramembranous and endochondral ossification [3, 4]. We then analyzed the dynamic expression patterns of the five most highly expressed miRNAs selected, from the microarray data, using real-time PCR on days 3, 7, 10, 14, 21, and 28. These time points were selected to represent specific physiological events during fracture healing, including inflammation, angiogenesis, chondrogenesis, and ossification, which corresponds to the inflammation phase to the end of the reparative phase [15, 19, 20]. As atrophic nonunion is established at 8 weeks after surgery in our unhealing fracture model [15], it would be interesting to compare the expression levels of the selected miRNAs between standard healing fractures and unhealing fractures and investigate their time-course changes at later time points such as weeks 6 and 8. In future studies, we will investigate their expression levels at later time points.

Of the five identified miRNAs, miR-140-3p, miR-181a-5p, and miR-451a have been reported to be involved in the regulation of inflammatory responses (Table 2). Inflammation is a critical factor during fracture healing, with inflammatory cells and molecular factors appearing locally at the fracture site in a distinct spatial and temporal manner [21]. During the initial inflammatory phase of fracture healing, inflammatory cells are rapidly recruited to the site of injury, neutrophils being the first cells to invade, followed by macrophages and lymphocytes. During the repair phase, osteal macrophages, which are present on the endosteal and periosteal surfaces, are pivotal for intramembranous ossification, whereas inflammatory macrophages recruited to a fracture site contribute to endochondral ossification. Highly-regulated inflammatory signaling during fracture healing is essential for priming bone regeneration. Disturbance to the finely-tuned inflammatory responses at the site of fracture has been shown to impair vascularization, reduce bone formation, disturb osteoclastic function, and consequently, lead to nonunions [21].

**Table 2:** Validated target genes of the five up-regulated miRNAs

miRNA	Target genes	References
miR-140-3p miR-140-5p miR-181a-5p miR-181d-5p miR-451a	NCOA1, NRIP1 Dnpep IL-1a, Bcl-2, TGFBI, TβR-I Bcl-2 14-3-3ζ, Rab5a, CUGBP2	22 29 24, 30 34 31 26 35

NCOA1, nuclear receptor coactivator 1; NRIP1, nuclear receptor-interacting protein 1; Dnpep, aspartyl aminopeptidase; IL-1a, interleukin-1 alpha; Bcl-2, B-cell lymphoma-2; TGFBI, TGF-β-induced; TβR-I, TGF-β type I receptor; CUGBP2, CUG triplet repeat-binding protein 2

miR-140-3p negatively regulates nuclear factor-κB (NF-κB) inflammatory signaling by regulating the expression of nuclear receptor coactivator 1 (NCOA1) and nuclear receptor-interacting protein 1 (NRIP1), both of which are NF-κB coactivators [22]. One of the primary molecular responses to tumor necrosis factor (TNF)-α signaling is activation of NF-κB [23]. In contrast, miR-181a-5p regulates inflammatory responses by directly targeting and down-regulating interleukin (IL)-1a [24]. Kon et al. demonstrated that in a mouse model of fracture healing, expression levels of TNF-α and IL-1a exhibited peaks in the inflammatory phase, declined to undetectable levels during the repair phase, and increased again in the remodeling phase [25]. This biphasic pattern is opposite to the monophasic expression pattern of miR-140-3p and miR-181a-5p observed in our study where the expression levels peaked in the repair phase (Fig. 2a, c), indicating that miR-140-3p and miR-181a-5p may suppress the expression of TNF-α and IL-1a, respectively, in the repair phase.

miR-451a reduces inflammation by suppressing phosphorylation of p38 mitogen activated protein kinase (MAPK) via 14-3-3ζ and Rab5a (Table 2) [26]. p38 MAPK plays an important signaling role in orchestrating injury or stress-induced responses and in bone formation [27]. Pro-inflammatory cytokines, such as TNF-α and IL-1a, activate p38 MAPK. In addition, activation and signaling of p38 MAPK also lead to the production of these inflammatory cytokines and their signal transduction. The interaction between p38 MAPK and pro-inflammatory cytokines is important in controlling life and death signaling cascades in osteoblasts and chondrocytes. Taken together, miR-140-3p, miR-181a-5p, and miR-451a may play important roles during fracture healing via the regulation of inflammation.

The process of fracture healing closely resembles normal skeletal development, which occurs by intramembranous and endochondral ossification [28]. Of the five identified miRNAs, miR-140-5p, miR-181a-5p, miR-181d-5p, and miR-451a have been reported to be involved in the regulation of skeletal development (Table 2). miR-140-5p is abundantly expressed in cartilaginous tissues during embryogenesis and also in adult cartilage [9]. Nakamura et al. demonstrated that miR-140-5p plays an essential role in skeletal development by regulating processes of endochondral ossification [29]. They identified Dnpep as a miR-140-5p target gene whose up-regulation plays a causal role in the skeletal defects of *MiR-140*-null mice by reducing BMP signaling.

miR-181a-5p and miR-181d-5p target B-cell lymphoma-2 (Bcl-2) [30, 31]. Bcl-2 proteins are family of cytosolic proteins involved in apoptotic pathways and regulate the “mitochondrial” pathway to apoptosis. Bcl-2 has been reported to be directly involved and required for skeletal development [32]. Bcl-2 is involved in the regulation of the programmed cell death of hypertrophic chondrocytes in the growth plate, an event that is critical for endochondral ossification. Furthermore, using osteoblast-specific *Bcl-2* transgenic mice, Moriishi et al. demonstrated that over-expression of Bcl-2 in osteoblasts inhibits their differentiation and induces osteocyte apoptosis [33]. Bhushan et al. demonstrated that miR-181a-5p was induced during different stages of mouse calvarial and tibial development, indicating a role in both endochondral and intramembranous ossification [34]. This study also demonstrated that miR-181a-5p promotes osteoblastic differentiation via repression of transforming growth factor-β (TGF-β) signaling molecules by targeting the negative regulators of osteoblastic differentiation, TGF-β-induced (TGFBI) and TGF-β type I receptor (TβR-I).

Finally, miR-451a targets CUG triplet repeat-binding protein 2 (CUGBP2), an RNA-binding protein that interacts with COX-2 mRNA 3′-UTR and inhibits its translation [35]. Up-regulation of miR-451a down-regulates CUGBP2, thereby significantly increasing COX2 protein levels. COX2 acts as a stress response and is responsible for high levels of prostaglandin production during inflammation. COX2 is an important factor partaking in chondrocyte hypertrophy during endochondral ossification in skeletal development [36]. In addition, COX2 is a critical regulator of fracture healing in animal models [37, 38]. Xie et al. showed that COX2 expression peaks at the early stages of intramembranous and endochondral repair, and is subsequently reduced during the remodeling phase [37]. Fractures in *Cox2*^−/−^ mice are deficient in reparative bone formation and exhibit persistent undifferentiated mesenchyme, suggesting that COX2 is necessary for normal fracture healing [38]. Zhang et al. concluded that COX2 was required for both intramembranous and endochondral ossification during fracture healing [38]. Collectively, these four miRNAs, namely miR-140-5p, miR-181a-5p, miR-181d-5p, and miR-451a, may play important roles during fracture healing by regulating intramembranous and/or endochondral ossification.

The present results have clinical implications. Fractures with high-energy injury, such as the presence of comminution, open fractures, massive bone defects, severe soft tissue damage, and local blood supply damage are at high risk of delayed union or nonunion [39]. Many biological and biophysical interventions have been used to attempt acceleration of fracture healing in such cases, including the use of exogenous growth factors, such as bone morphogenetic proteins, low-intensity pulsed ultrasound, and stem/progenitor cell transplantation [40–42]. Although most of these strategies exhibit relatively satisfactory results, there are some notable limitations to their effectiveness and availability. The recent discovery of miRNAs and their ability to regulate global gene expression patterns in a variety of tissues and processes suggests potential therapeutic strategies involving targeting of miRNAs. Several therapeutic trials targeting miRNAs have been conducted [43]. Recently, Miravirsen (SPC3649) entered human clinical trials for the treatment of hepatitis C virus (HCV) infection as the first miRNA-targeted drug. Janssen et al. reported phase 2a clinical trial results showing that Miravirsen, which sequesters miR-122, dose-dependently and sustainably decreased HCV RNA levels in patients with chronic HCV infection [44]. The miRNAs identified in the current study might represent key tools for the development of molecular therapies to enhance fracture healing. For example, in fracture patients at high risk for delayed union or nonunion, local administration of synthesized miRNA oligonucleotides to the fracture site may accelerate fracture healing. However, further *in vivo* functional analyses will be required to define the precise role of each miRNA during fracture healing.

## Conclusions

The five miRNAs, miR-140-3p, miR-140-5p, miR-181a-5p, miR-181d-5p, and miR-451a, identified using microarray and real-time PCR analyses may play important roles during fracture healing. Our findings provide valuable information to further understand the molecular mechanisms of fracture healing and may lead to the development of miRNA-based tissue engineering strategies to promote fracture healing and bone regeneration.

## References

[1] United States Bone and Joint Initiative. The Burden of Musculoskeletal Diseases in the United States (BMUS), Third Edition. 2014. http://www.boneandjointburden.org. Accessed 5 July 2015.

[2] Rodriguez-Merchan EC, Forriol F (2004). Nonunion: general principles and experimental data. Clin Orthop Relat Res.

[3] Gerstenfeld LC, Cullinane DM, Barnes GL, Graves DT, Einhorn TA (2003). Fracture healing as a post-natal developmental process: molecular, spatial, and temporal aspects of its regulation. J Cell Biochem.

[4] Dimitriou R, Tsiridis E, Giannoudis PV (2005). Current concepts of molecular aspects of bone healing. Injury.

[5] Rundle CH, Wang H, Yu H, Chadwick RB, Davis EI, Wergedal JE (2006). Microarray analysis of gene expression during the inflammation and endochondral bone formation stages of rat femur fracture repair. Bone.

[6] Bartel DP (2004). MicroRNAs: genomics, biogenesis, mechanism, and function. Cell.

[7] Zhang B, Wang Q, Pan X (2007). MicroRNAs and their regulatory roles in animals and plants. J Cell Physiol.

[8] Lu J, Getz G, Miska EA, Alvarez-Saavedra E, Lamb J, Peck D (2005). MicroRNA expression profiles classify human cancers. Nature.

[9] Hong E, Reddi AH (2012). MicroRNAs in chondrogenesis, articular cartilage, and osteoarthritis: implications for tissue engineering. Tissue Eng Part B Rev.

[10] van Wijnen AJ, van de Peppel J, van Leeuwen JP, Lian JB, Stein GS, Westendorf JJ (2013). MicroRNA functions in osteogenesis and dysfunctions in osteoporosis. Curr Osteoporos Rep.

[11] Garmilla-Ezquerra P, Sañudo C, Delgado-Calle J, Pérez-Nuñez MI, Sumillera M, Riancho JA (2015). Analysis of the bone microRNome in osteoporotic fractures. Calcif Tissue Int.

[12] Murata K, Ito H, Yoshitomi H, Yamamoto K, Fukuda A, Yoshikawa J (2014). Inhibition of miR-92a enhances fracture healing via promoting angiogenesis in a model of stabilized fracture in young mice. J Bone Miner Res.

[13] Seeliger C, Karpinski K, Haug AT, Vester H, Schmitt A, Bauer JS (2014). Five freely circulating miRNAs and bone tissue miRNAs are associated with osteoporotic fractures. J Bone Miner Res.

[14] Waki T, Lee SY, Niikura T, Iwakura T, Dogaki Y, Okumachi E (2015). Profiling microRNA expression in fracture nonunions: Potential role of microRNAs in nonunion formation studied in a rat model. Bone Joint J.

[15] Bonnarens F, Einhorn TA (1984). Production of a standard closed fracture in laboratory animal bone. J Orthop Res.

[16] Kokubu T, Hak DJ, Hazelwood SJ, Reddi AH (2003). Development of an atrophic nonunion model and comparison to a closed healing fracture in rat femur. J Orthop Res.

[17] Koh A, Niikura T, Lee SY, Oe K, Koga T, Dogaki Y (2011). Differential gene expression and immunolocalization of insulin-like growth factors and insulin-like growth factor binding proteins between experimental nonunions and standard healing fractures. J Orthop Res.

[18] Niikura T, Hak DJ, Reddi AH (2006). Global gene profiling reveals a downregulation of BMP gene expression in experimental atrophic nonunions compared to standard healing fractures. J Orthop Res.

[19] Einhorn TA (1998). The cell and molecular biology of fracture healing. Clin Orthop Relat Res.

[20] Phillips AM (2005). Overview of the fracture healing cascade. Injury.

[21] Claes L, Recknagel S, Ignatius A (2012). Fracture healing under healthy and inflammatory conditions. Nat Rev Rheumatol.

[22] Takata A, Otsuka M, Kojima K, Yoshikawa T, Kishikawa T, Yoshida H (2011). MicroRNA-22 and microRNA-140 suppress NF-κB activity by regulating the expression of NF-κB coactivators. Biochem Biophys Res Commun.

[23] Kanegae Y, Tavares AT, Izpisúa Belmonte JC (1998). Verma IM Role of Rel/NF-kappaB transcription factors during the outgrowth of the vertebrate limb. Nature.

[24] Xie W, Li M, Xu N, Lv Q, Huang N, He J (2013). MiR-181a regulates inflammation responses in monocytes and macrophages. PLoS One.

[25] Kon T, Cho TJ, Aizawa T, Yamazaki M, Nooh N, Graves D (2001). Expression of osteoprotegerin, receptor activator of NF-kappaB ligand (osteoprotegerin ligand) and related proinflammatory cytokines during fracture healing. J Bone Miner Res.

[26] Murata K, Yoshitomi H, Furu M, Ishikawa M, Shibuya H, Ito H (2014). MicroRNA-451 down-regulates neutrophil chemotaxis via p38 MAPK. Arthritis Rheumatol.

[27] Zhou FH, Foster BK, Zhou XF, Cowin AJ, Xian CJ (2006). TNF-alpha mediates p38 MAP kinase activation and negatively regulates bone formation at the injured growth plate in rats. J Bone Miner Res.

[28] Ferguson C, Alpern E, Miclau T, Helms JA (1999). Does adult fracture repair recapitulate embryonic skeletal formation?. Mech Dev.

[29] Nakamura Y, Inloes JB, Katagiri T, Kobayashi T (2011). Chondrocyte-specific microRNA-140 regulates endochondral bone development and targets Dnpep to modulate bone morphogenetic protein signaling. Mol Cell Biol.

[30] Ouyang YB, Lu Y, Yue S, Giffard RG (2012). miR-181 targets multiple Bcl-2 family members and influences apoptosis and mitochondrial function in astrocytes. Mitochondrion.

[31] Wang XF, Shi ZM, Wang XR, Cao L, Wang YY, Zhang JX (2012). MiR-181d acts as a tumor suppressor in glioma by targeting K-ras and Bcl-2. J Cancer Res Clin Oncol.

[32] Amling M, Neff L, Tanaka S, Inoue D, Kuida K, Weir E (1997). Bcl-2 lies downstream of parathyroid hormone-related peptide in a signaling pathway that regulates chondrocyte maturation during skeletal development. J Cell Biol.

[33] Moriishi T, Maruyama Z, Fukuyama R, Ito M, Miyazaki T, Kitaura H (2011). Overexpression of Bcl2 in osteoblasts inhibits osteoblast differentiation and induces osteocyte apoptosis. PLoS One.

[34] Bhushan R, Grünhagen J, Becker J, Robinson PN, Ott CE, Knaus P (2013). miR-181a promotes osteoblastic differentiation through repression of TGF-β signaling molecules. Int J Biochem Cell Biol.

[35] Zhang X, Wang X, Zhu H, Zhu C, Wang Y, Pu WT (2010). Synergistic effects of the GATA-4-mediated miR-144/451 cluster in protection against simulated ischemia/reperfusion-induced cardiomyocyte death. J Mol Cell Cardiol.

[36] Welting TJ, Caron MM, Emans PJ, Janssen MP, Sanen K, Coolsen MM (2011). Inhibition of cyclooxygenase-2 impacts chondrocyte hypertrophic differentiation during endochondral ossification. Eur Cell Mater.

[37] Xie C, Liang B, Xue M, Lin AS, Loiselle A, Schwarz EM (2009). Rescue of impaired fracture healing in COX-2^−/−^ mice via activation of prostaglandin E2 receptor subtype 4. Am J Pathol.

[38] Zhang X, Schwarz EM, Young DA, Puzas JE, Rosier RN, O’Keefe RJ (2002). Cyclooxygenase-2 regulates mesenchymal cell differentiation into the osteoblast lineage and is critically involved in bone repair. J Clin Invest.

[39] Niikura T, Lee SY, Sakai Y, Nishida K, Kuroda R, Kurosaka M (2014). Causative factors of fracture nonunion: the proportions of mechanical, biological, patient-dependent, and patient-independent factors. J Orthop Sci.

[40] Reddi AH (2001). Bone morphogenetic proteins: from basic science to clinical applications. J Bone Joint Surg Am.

[41] Rubin C, Bolander M, Ryaby JP, Hadjiargyrou M (2001). The use of low-intensity ultrasound to accelerate the healing of fractures. J Bone Joint Surg Am.

[42] Arthur A, Zannettino A, Gronthos S (2009). The therapeutic applications of multipotential mesenchymal/stromal stem cells in skeletal tissue repair. J Cell Physiol.

[43] Soifer HS, Rossi JJ, Saetrom P (2007). MicroRNAs in disease and potential therapeutic applications. Mol Ther.

[44] Janssen HL, Kauppinen S, Hodges MR (2013). HCV infection and miravirsen. N Engl J Med.

